# Supplementary material for: TiO2-Modified Montmorillonite-Supported Porous Carbon-Immobilized Pd Species Nanocomposite as an Efficient Catalyst for Sonogashira Reactions
Source: Molecules. 2023 Mar 6;28(5):2399. doi: 10.3390/molecules28052399 (PMC10005427; doi:10.3390/molecules28052399)
Supplement: Supplementary file 1 [file molecules-28-02399-s001.zip › molecules-2225196-supplementary.pdf]

Supporting Information

TiO_2 modified montmorillonite supported porous carbon im-mobilized Pd species nanocomposite as an efficient catalyst for Sonogashira reactions

Yuli Chen [1], Kailang Sun [1], Taojun Zhang [1], Jie Zhou [1], Yonghong Liu [1], Minfeng Zeng [1*], Xiaorong Ren [1], Ruokun Feng [1], Zhen Yang [1*], Peng Zhang [2], Baoyi Wang [2], Xingzhong Cao[2*]

[1] Research Center of Advanced Catalytic Materials & Functional Molecular Synthesis, Zhejiang Key Laboratory of Alternative Technologies for Fine Chemicals Process, College of Chemistry & Chemical Engineering, Shaoxing University, Shaoxing 312000, China;

[2] Institute of High Energy Physics, The Chinese Academy of Science, Beijing 100049, China;

* Correspondence: zengmf@usx.edu.cn (M. Z.); yangzhen09@usx.edu.cn (Z. Y.); caoxzh@ihep.ac.cn (X. C.)

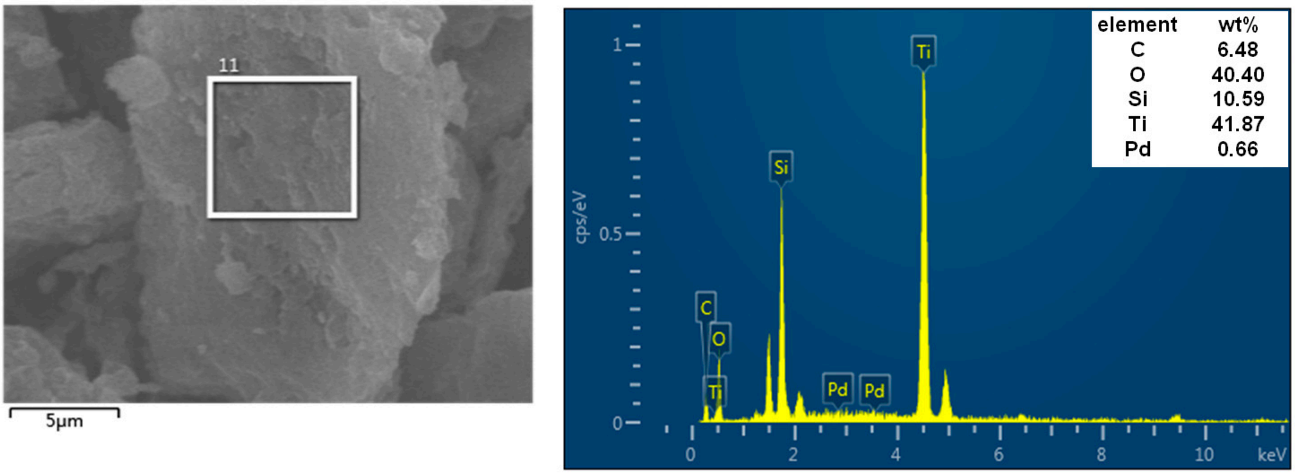

Figure S1. SEM-EDX scanning results of the TiO_2-MMT_{60}/PCN_{40}@Pd^0 nanocomposites.

SEM-EDX tests was performed with scanning electron microscope (JEM-6360, JEOL Ltd. Japan) equipped with an energy dispersive X ray-spectroscopy (Oxford EDX system).

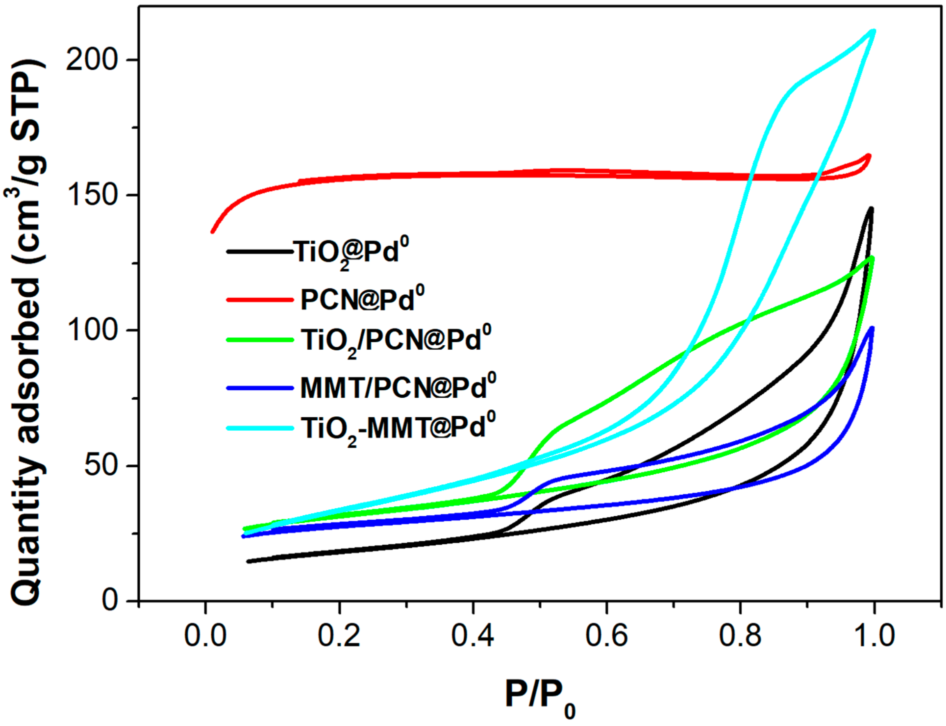

Figure S2. N_2 adsorption/desorption isotherms the heterogeneous catalysts.

Table S1. Structure parameter of heterogeneous catalysts extracted from the isotherms in **Figure S2**.

**Sample**	S_{BET} **(m²/g)**	A_{mic} **(m²/g)**	V_{tot} **(cm³/g)**
TiO_2-Pd^0	63.2	23.9	0.21
PCN-Pd^0	521.9	453.9	0.24
TiO_2/PCN-Pd^0	103.7	53.5	0.19
MMT/PCN-Pd^0	89.4	48.1	0.14
TiO_2-MMT-Pd^0	121.8	34.0	0.33

S_{BET}: specific surface area, by Brumanuer-Emmet-Teller method; A_{mic}: micropores area obtained by *t*-Polt method; V_{tot}: total volume of pores, the N_2 quantity absorbed at a relative pressure of P/P_0=0.99.

Table S2. Model Sonogashira coupling reaction between iodo benzene and phenyl acetylene catalyzed by the heterogeneous catalysts.

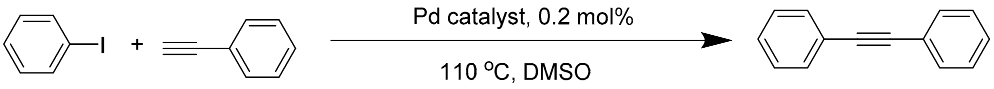

Entry	Catalyst	Yield (%)
1	no catalyst	no product
2	TiO_2@Pd^0	95
3	PCN@Pd^0	98
4	TiO_2/PCN@Pd^0	98
5	MMT/PCN@Pd^0	97
6	TiO_2-MMT@Pd^0	96
7	TiO_2-MMT_{60}/PCN_{40}@Pd^0	99

[a] GC/MS yield

The preparation process of the heterogeneous catalysts is as follows:

TiO_2@Pd^0: 2 g of nano TiO_2 particles (sized about10 nm) were added into the 20 mL of Na_2PdCl_4 solution. Na_2PdCl_4 stock solution was made by dissolving 0.3 g of $PdCl_2$ and 2 g of NaCl in 100 mL of deionized water. The resulting mixture was allowed to stir at room temperature until the solution turned from brown to colorless. The collected powders were then washed until free of chloride anion, followed by reduction in ethylene glycol at 80 °C for 1 h.

PCN@Pd^0: 1.33g of CS was dissolved in 100 mL of 2 wt% CH_3COOH solution. 5 mL of Na_2PdCl_4 solution (contain 0.09 mmol of Pd) was dropped into the CS solution. Then, the CS-Pd^{2+} mixture solution were dried at 60 °C to form blend membranes. Then the blend membrane was carbonized at 800 °C for 4 h under N_2 atmosphere in the tubular muffle furnace. The collected powders were then washed with deionized water, followed by reduction in ethylene glycol at 80 °C for 1 h.

TiO_2/PCN@Pd^0: 2 g of nano TiO_2 particles (sized about10 nm) were added into the above CS-Pd^{2+} mixture solution. Then, the solution was continuously stirred at 60 °C (water bath heating) for 10 h. The TiO_2/CS@Pd^{2+} was separated from the suspension by centrifugation. After washing with deionized water to neutral and naturally drying, the TiO_2/CS@Pd^{2+} were carbonized at 800 °C for 4 h under N2 atmosphere in the tubular muffle furnace. The collected powders were then washed with deionized water, followed by reduction in ethylene glycol at 80 °C for 1 h.

MMT/PCN@Pd^0: 2 g of nano MMT were added into the above CS-Pd^{2+} mixture solution. Then, the solution was continuously stirred at 60 °C (water bath heating) for 10 h. The MMT/CS@Pd^{2+} was separated from the suspension by centrifugation. After washing with deionized water to neutral and naturally drying, the MMT/CS@Pd^{2+} were carbonized at 800 °C for 4 h under N_2 atmosphere in the tubular muffle furnace. The collected powders were then washed with deionized water, followed by reduction in ethylene glycol at 80 ℃ for 1 h.

TiO_2-MMT@Pd^0: 2 g of nano TiO_2-MMT particles (sized about10 nm) were added into the 20 mL of Na_2PdCl_4

solution. Na_2PdCl_4 stock solution was made by dissolving 0.3 g of $PdCl_2$ and 2 g of NaCl in 100 mL of deionized water. The resulting mixture was allowed to stir at room temperature until the solution turned from brown to colorless. The collected powders were then washed until free of chloride anion, followed by reduction in ethylene glycol at 80 °C for 1 h.

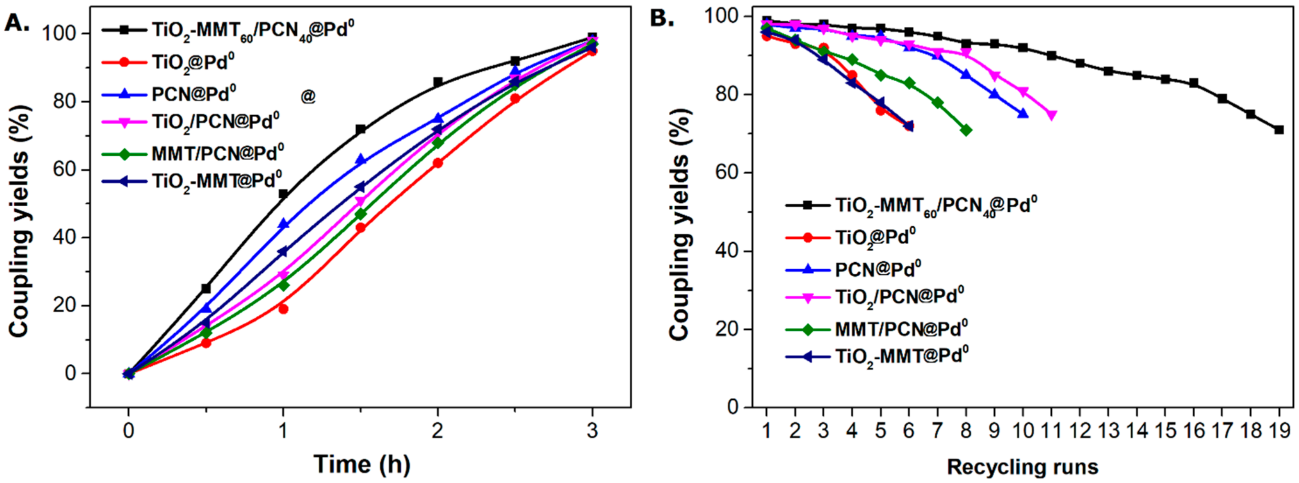

Figure S3. Performances of the heterogeneous catalysts applied in model Sonogashira reaction: **A.** coupling yields vs time; **B.** coupling yields vs recycling runs.

Table S3. A comparison of TiO_2-MMT/PCN@Pd^0 catalyst with recent reported catalysts for Sonogashira reactions.

Entry	Catalyst	Metal (mol%)	Number of cycles	Reaction conditions	Ref.
1	*h*-Fe_3O_4@TiO_2-NH_2/Pd	0.18%	5	DMF, 90 °C, 3 h	75
2	TiO_2@ Pd (II)[PATA-NH_2]	0.35%	5	H_2O, 3 h, r.t.	76
3	K10@Pd(II)APTES	0.3%	4	DMF, 7 h, 80°C	77
4	MMT/CS@Pd, Cu	1%	6	PPh_3, H_2O/DME, 8 h, 80 °C	78
5	CHT@Pd	0.23%	4	DMF, 10 h, 80 °C	79
6	Hal-pDA-NPC@Pd	0.5%	10	H_2O, 50 min, r.t.	80
7	TiO_2-MMT_{80}/PCN_{20}-Pd^0	0.2%	16	DMSO, 3 h, 110°C	this work
8	TiO_2-MMT_{60}/PCN_{40}-Pd^0	0.2%	19	DMSO, 3 h, 100 °C	this work

^{1}H NMR of the coupling products

Diphenylacetylene ^{1}H NMR (400 MHz, Chloroform-*d*) δ 7.79 – 7.44 (m, 4H), 7.32 (dd, *J* = 5.1, 2.1Hz,6H).

1-Methyl-2-phenylthynyl-benzene ^{1}H NMR (400 MHz, Chloroform-*d*) δ 7.59 – 7.44 (m, 2H), 7.44 – 7.24 (m, 5H), 7.20 (t, *J* = 7.6 Hz, 1H), 7.11 (d, *J* = 7.6 Hz, 1H), 2.31 (s, 3H).

1-Methyl-3-phenylthynyl-benzene ^{1}H NMR (400 MHz, Chloroform-*d*) δ 7.58 – 7.50 (m, 2H), 7.49 (dt, *J* = 7.4, 1.1 Hz, 1H), 7.42 – 7.23 (m, 3H), 7.23 – 7.17 (m, 2H), 7.17 – 7.08 (m, 1H), 2.50 (s, 3H).

1-Methyl-4-phenylethynyl benzene 1HNMR (400 MHz, Chloroform-*d*) δ 7.67 – 7.62 (m, 2H), 7.58 – 7.53 (m, 2H), 7.48 – 7.38 (m, 3H), 7.25 (d, J = 7.9Hz, 2H), 2.46 (s, 3H).

1-Chloro-2-phenylthynyl-benzene ^{1}H NMR (400 MHz, Chloroform-*d*) δ 7.59-7.50 (m, 3H), 7.40-7.36 (m, 1H), 7.31 (qd, J = 3.7, 1.5 Hz, 3H), 7.23-7.15 (m, 2H).

1-Bromo-3-phenylthynyl-benzene ^{1}H NMR (400 MHz, Chloroform-*d*) δ 7.67 (t, *J* = 1.8 Hz, 1H), 7.56 – 7.47 (m, 2H), 7.48 – 7.38 (m, 2H), 7.38 – 7.26 (m, 3H), 7.25 – 7.11 (m, 1H).

1-Fluoro-4-phenylthynyl-benzene ^{1}H NMR (400 MHz, Chloroform-*d*) δ 7.80 – 7.39 (m, 4H), 7.39 – 7.09 (m, 3H), 7.09 – 6.85 (m, 2H).

1-Methoxy-3-phenylthynyl-benzene ^{1}H NMR (400 MHz, Chloroform-*d*) δ 7.74-7.57 (m, 2H), 7.41 (qd, J = 4.9, 1.7 Hz, 3H), 7.32 (t, J = 7.9 Hz, 1H), 7.23 (dt, J = 7.6, 1.3 Hz, 1H), 7.16 (dd, J = 2.5, 1.4 Hz, 1H), 6.96 (dt, J = 8.3, 1.7 Hz, 1H), 3.86 (s, 3H).

1-Methoxy-4-(phenylethynyl)benzene ^{1}HNMR (400 MHz, Chloroform-*d*) δ 7.67 – 7.62 (m, 2H), 7.58 – 7.53 (m, 2H), 7.48 – 7.38 (m, 3H), 7.25 (d, J = 7.9 Hz, 2H), 2.46 (s, 3H)

1-Fluoro-3-phenylthynyl-benzene ^{1}H NMR (400 MHz, Chloroform-*d*) δ 7.52 (dd, J = 6.7, 3.1 Hz, 2H), 7.34 (q, J = 3.0 Hz, 3H), 7.28 (dd, J = 6.9, 4.4 Hz, 2H), 7.25 – 7.19 (m, 1H), 7.02 (ddt, J = 9.3, 4.4, 2.6 Hz, 1H)

1-Phenylethynyl-naphthalene ^{1}H NMR (400 MHz, Chloroform-*d*) δ 8.49 (dd, J = 8.3, 1.2 Hz, 1H), 7.89 (ddt, J = 9.5, 8.4, 1.0 Hz, 2H), 7.81 (dd, J = 7.2, 1.2 Hz, 1H), 7.76 – 7.54 (m, 4H), 7.54 – 7.36 (m, 4H).

4-Phenylethynyl-9H-fluorene ^{1}H NMR (400 MHz, Chloroform-*d*) δ 7.90 – 7.70 (m, 3H), 7.69 – 7.50 (m, 4H), 7.39 (dt, *J* = 15.2, 7.6 Hz, 5H), 3.94 (s, 2H).
